# Maximal haemostatic effect is attained in porcine skin within 7 min of the administration of a local anaesthetic together with epinephrine, refuting the need for a 30 min waiting time

**DOI:** 10.1016/j.jpra.2018.12.005

**Published:** 2018-12-22

**Authors:** Rafi Sheikh, Jenny Hult, Josefine Bunke, Ulf Dahlstrand, Cu Ansson, Khashayar Memarzadeh, Malin Malmsjö

**Affiliations:** Lund University, Skane University Hospital, Department of Clinical Sciences Lund, Ophthalmology, Lund, Sweden, Ögonklinik A, admin, 2nd floor, Kioskgatan 1B, SE-221 85 Lund, Sweden

**Keywords:** Local anaesthesia, Haemostasis, Pig, Skin

## Abstract

**Objective:**

Based on clinical experience gained over many years, the maximal haemostatic effect following administration of local anaesthetics containing epinephrine is generally believed to occur within 10 min. Surprisingly, it was found in a recent study, in which bleeding was quantified intraoperatively, that maximal haemostasis did not occur until 30 min. If this is indeed the case, then it would be necessary to extend the preoperative waiting time to minimize perioperative bleeding.

We have carried out a carefully controlled study on the time delay between administration of a local anaesthetic containing epinephrine and maximal haemostasis in a surgical setting.

**Methods:**

Lidocaine 20 mg/ml (2%) or lidocaine + epinephrine 12.5 µg/ml (1:80,000) was injected into the skin of eight pig flanks. Bleeding was induced after 3, 5, 7, 15 and 30 min by making a 10 mm incision at each injection site. Blood was collected for 1 min and weighed.

**Results:**

A gradual reduction in bleeding was observed, with maximal reduction after only 7 min (54%, *p* < 0.05, 95% CI: 44–64%). No further significant reduction in bleeding was observed (62% at 15 and 66% at 30 min, *p* = n.s. compared to 7 min).

**Conclusions:**

Maximal haemostatic effect in the current setting was observed within 7 min of injection of lidocaine with epinephrine. This is in good agreement with previous empirical findings, and we see no reason to prolong the preoperative waiting time.

## Introduction

Epinephrine has been used in local anaesthetics to reduce perioperative bleeding since 1903^1^; nevertheless, the time before maximal haemostasis is achieved has recently become the subject of debate. To achieve the best possible surgical result, it is of great importance that the surgeon knows how long to wait before making the first incision, while at the same time avoiding patient distress caused by long waiting times. Employing the optimal time delay between the administration of local anaesthetics and skin incision also increases health care efficiency. Textbooks often recommend a time delay of 10 min^2^; which is also commonly used in clinical practice. However, using spectroscopy in the arm skin of healthy volunteers, McKee et al. reported that the lowest haemoglobin level was seen after 26 min.^3^ In a later study, they measured bleeding in skin during carpal tunnel surgery, and found a significant decrease after 30 min.^4^ No studies have been carried out in which the amount of bleeding has been determined quantitatively at many different times following the administration of local anaesthetics with epinephrine. In the present study, we have measured the amount of bleeding resulting from a surgical incision in the pig flank, following the injection of lidocaine only (20 mg/ml, 2%) and lidocaine + epinephrine (12.5 µg/ml, 1:80,000), after waiting 3, 5, 7, 15 and 30 min.

## Methods

### Animals and anaesthesia

Eight pigs, with a bodyweight of 70 kg were fasted overnight and induced with anaesthesia, by intravenous sodium thiopental (Pentothal®; Abbot Scandinavia, Stockholm, Sweden; 4 mg/kg) and fentanyl (Leptanal®; Lilly, France; 2 µg/kg), and allowed to stabilize for 1 h before the experiments started.^5^

### Surgical procedure

**Figure 1:**
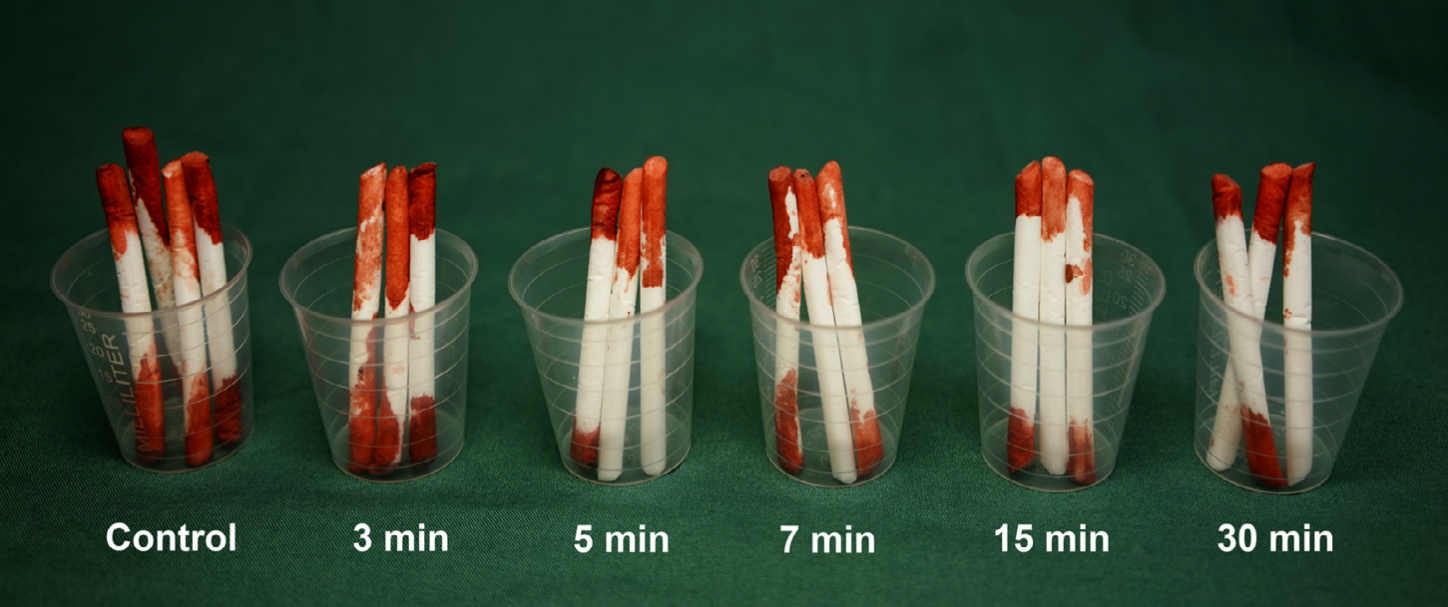
Representative example of surgical swabs with blood collected from the lidocaine (control) and lidocaine + epinephrine injection sites following incision, after 3, 5, 7, 15 and 30 min.

Lidocaine (Lidokain®; Mylan Hospital AS, Oslo, Norway; 20 mg/ml), denoted ‘control’, and lidocaine + epinephrine (Xylocain Dental® Adrenalin, 20 mg/ml lidocaine + 12.5 µg/ml epinephrine, Dentsply Ltd., York, PA, USA), denoted ‘epinephrine’, were preheated to 38 °C in a water bath. Multiple sites were marked, 5 cm apart, on the pig flank, and subcutaneous bolus injections were performed with 1.0 ml of local anaesthetic with or without epinephrine. A square-shaped excision of 10 × 10 mm skin down to subcutis was made using a scalpel, after 1 min at the control site, and after 3, 5, 7, 15 and 30 min at the epinephrine sites. Viscose nonwoven surgical swabs (ocular sticks, Pro-Ophta®, Lohmann & Rauscher GmbH & Co. KG, Rengsdorf, Germany) were used to collect blood for 1 min immediately after incision at all sites (see Figure 1). The swabs were placed in pill cups and weighed using calibrated scales (Ohaus Scout, model STX223, OHAUS Corporation, Parsippany, NJ, USA). The weight of blood was obtained by subtracting the weight of the clean swabs and pill cups.

### Calculations and statistics

Eight pigs were used in this study and multiple experiments were performed in each pig. Bleeding is expressed as the weight of blood (mg), and the reduction in bleeding as a result of epinephrine was calculated as the % of control and expressed as mean values and 95% confidence intervals (95% CI). The results are given in scatter plots, with a line through the mean values. Statistical analysis was performed using the Kruskal–Wallis test with Dunn's post-hoc test for multiple comparisons. Significance was defined as *p* < 0.05, and *p* > 0.05 (not significant, n.s.). Calculations were performed using GraphPad Prism 7.0a (GraphPad software Inc., San Diego, CA, USA).

## Results

### Bleeding

**Figure 2:**
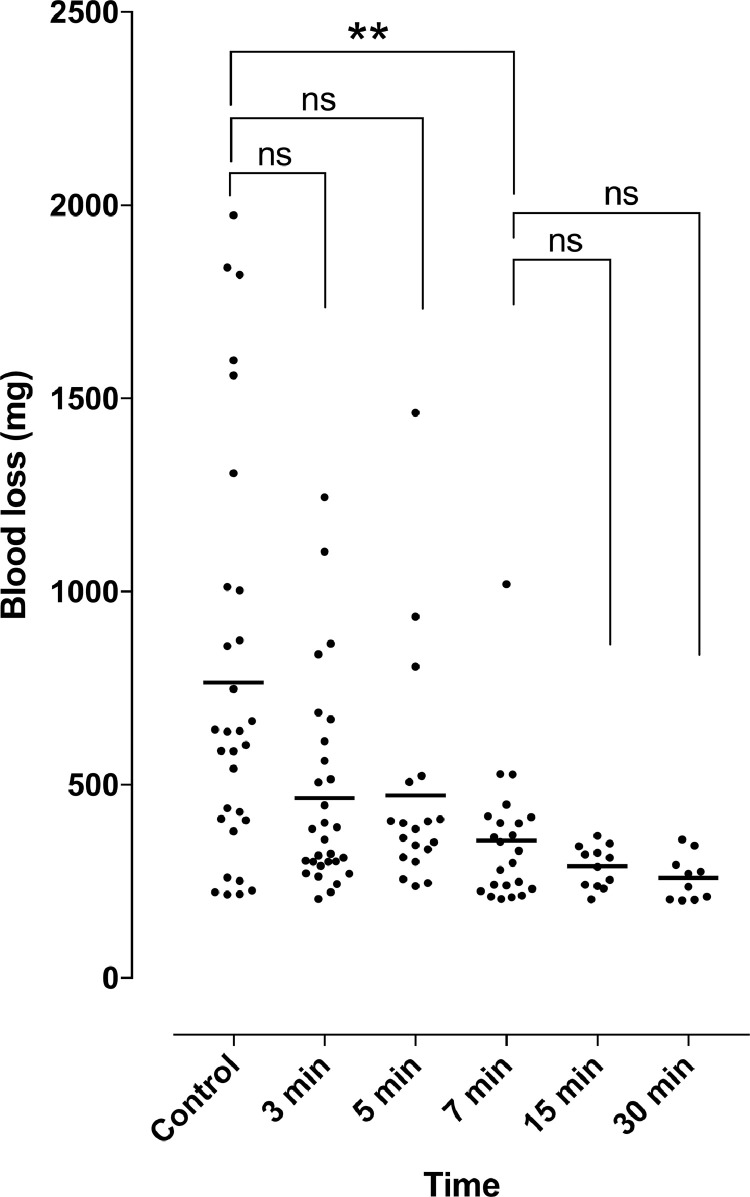
Blood loss (mg) after the injection of lidocaine only (control) and lidocaine + epinephrine at 3, 5, 7, 15 and 30 min.

Profuse bleeding was observed upon skin incision at the control site: mean weight 765 mg (216–1974 mg) 1 min after skin incision (*n* = 30). Bleeding decreased gradually with waiting time after the injection of epinephrine; being 61% of control after 3 min (*n* = 29, mean weight 466 mg (205–1245 mg), *p* = n.s), 62% after 5 min (*n* = 19, mean weight 473 (238–1464 mg), *p* = n.s.) and 46% after 7 min (*n* = 23, mean weight 355 (205–1020 mg), *p* < 0.05, 95% CI: 36–56%). No further decrease in bleeding was seen after 15 or 30 min (*p* = n.s.). Figure 2 shows detailed results regarding blood loss.

## Discussion

We have made quantitative measurements of bleeding 3, 5, 7, 15 and 30 min after the administration of a local anaesthetic containing epinephrine. The results showed a gradual reduction in bleeding during the first 7 min after injection, by which time the bleeding had decreased by 54%. No statistically significant decrease in bleeding was observed after 7 min. This is in good agreement with empirical knowledge and the general practice of waiting 7–10 min,^2^ and in direct contrast to the results presented by McKee et al., where a waiting time of 30 min was required to achieve maximal haemostasis.^3^, ^4^

There is a noted discrepancy between the results of this study and the work done by Mckee et al., but there are some differences in the experimental methodology. Mckee et al. used micropipettes, while we used weighing off surgical swabs for blood loss quantification. Furthermore, we performed our study dorsal porcine skin, while McKees study was performed on human forearms. The differences in species and skin location, and thereby the vasculature, may explain the discrepancy in the results. Furthermore, we have recently performed similar studies on eyelids in humans undergoing blepharoplasty, although less detailed and at fewer waiting times: 7, 15 and 30 min. The results of this study are in line with the present study, showing that the maximal haemostatic effect occurred within 7 min following the administration of lidocaine + epinephrine, also measured by quantifying blood loss.^6^

Studies have also been carried out in which perfusion was measured using laser-based techniques following the injection of local anaesthetics with epinephrine. Larrabee et al. used laser Doppler flowmetry (LDF) to measure perfusion in the skin of piglet trunks, and found maximal vasoconstriction to occur at 5–7 min.^7^ O'Malley et al. also used LDF, and found maximal vasoconstriction to occur after 3–4 min in the neck of patients undergoing head and neck surgery.^8^ Ghali et al. measured the blood flow in the forearm and face of healthy volunteers using LDF, and found the maximal decrease to occur after 8 min in the face, and 10 min in the forearm.^9^ In a study by our group on porcine eyelid flaps, where we used laser speckle contrast imaging to measure blood perfusion, we found that the time to maximal hypoperfusion after the injection of lidocaine + epinephrine was 75 s.^5^ In a similar study on porcine flank flaps, using LDF and laser speckle contrast imaging, we observed maximal hypoperfusion after approximately 120 s.^10^ The results presented above all support the occurrence of maximal haemostasis within 7–10 min.

In conclusion, we found that the injection of lidocaine with epinephrine induces maximal haemostasis within 7 min in pigs. Assuming that epinephrine exerts the same effect in humans, this confirms that the common clinical practice of waiting 10 min before surgical incision is appropriate and challenges the results of studies where a 30 min delay has been reported.^3^, ^4^

## Conflict of interest

None.

## Funding

This study was supported by the Swedish Government Grant for Clinical Research (ALF), the Skåne University Hospital (SUS) Research Grants, Crown Princess Margaret's Foundation (KMA), the Foundation for the Visually Impaired in the County of Malmöhus, The Nordmark Foundation for Eye Diseases at Skåne University Hospital, the Diabetes Society of South-West Skåne, and the Swedish Eye Foundation.
